# Markers of Systemic Inflammation and Apo-AI Containing HDL Subpopulations in Women with and without Diabetes

**DOI:** 10.1155/2014/607924

**Published:** 2014-09-02

**Authors:** Giuseppina T. Russo, Annalisa Giandalia, Elisabetta L. Romeo, Angela Alibrandi, Katalin V. Horvath, Bela F. Asztalos, Domenico Cucinotta

**Affiliations:** ^1^Department of Clinical and Experimental Medicine, University of Messina, Via C. Valeria, 98124 Messina, Italy; ^2^Department of Economical, Business and Environmental Sciences and Quantitative Methods, University of Messina, Piazza Pugliatti 1, 98122 Messina, Italy; ^3^Lipid Metabolism Laboratory, JM-USDA-Human Nutrition Research Center on Aging, Tufts University, 711 Washington Street, Boston, MA 02111, USA

## Abstract

*Background*. Besides their role in reverse cholesterol transport, HDL particles may affect the atherosclerotic process through the modulation of subclinical inflammation. HDL particles differ in size, composition, and, probably, anti-inflammatory properties. This hypothesis has never been explored in diabetic women, frequently having dysfunctional HDL. The potential relationship between lipid profile, Apo-AI containing HDL subclasses distribution, and common inflammatory markers (hsCRP, IL-6) was examined in 160 coronary heart disease- (CHD-) free women with and without type 2 diabetes. *Results*. Compared to controls, diabetic women showed lower levels of the atheroprotective large *α*-1, *α*-2, and pre-*α*-1 and higher concentration of the small, lipid-poor *α*-3 HDL particles (*P* < 0.05 all); diabetic women also had higher hsCRP and IL-6 serum levels (age- and BMI-adjusted *P* < 0.001). Overall, HDL subclasses significantly correlated with inflammatory markers: hsCRP inversely correlated with *α*-1 (*P* = 0.01) and pre-*α*-1 (*P* = 0.003); IL-6 inversely correlated with *α*-1 (*P* = 0.003), *α*-2 (*P* = 0.004), and pre-*α*-1 (*P* = 0.002) and positively with *α*-3 HDL (*P* = 0.03). Similar correlations were confirmed at univariate regression analysis. *Conclusions*. More atheroprotective HDL subclasses are associated with lower levels of inflammatory markers, especially in diabetic women. These data suggest that different HDL subclasses may influence CHD risk also through the modulation of inflammation.

## 1. Introduction

Cardiovascular disease is the primary cause of death also in diabetic women [1–4].

Low plasma levels of high-density lipoprotein cholesterol (HDL-C) have been largely recognized as a risk factor for coronary heart disease (CHD) [5, 6] and they are a common feature of insulin resistance states [7].

HDL class comprises very heterogeneous particles that can be separated by different methods, including two-dimensional gel electrophoresis that separates Apo-AI containing HDL particles according to their size and lipid content [5]. Specific particles have been shown to differently promote cholesterol efflux, suggesting a distinct role in reverse cholesterol transport (RCT) and CVD risk protection [5, 8–10]. Thus, Cheung et al. reported that the presence of CHD was more strongly associated with HDL particle size distribution than with low HDL-C level [11]. Furthermore, it was documented that low levels of *α*-1 and *α*-2 HDL particles were better predictors of CHD risk than total HDL-C concentration in both the Framingham Offspring Study and the VA-HIT study [12, 13].

We have recently shown that type 2 diabetes determines a shift in the distribution of HDL particles; in particular, when assessing HDL subpopulation distribution by two-dimensional gel electrophoresis, diabetic women had HDL that are selectively depleted in the large lipid-rich *α*-1, *α*-2, and pre-*α*-1 and enriched in the small, lipid-poor *α*-3 HDL subpopulations, resulting in HDL particles that were smaller in size and poor in cholesterol compared with those of unaffected subjects; this profile resembled that of men with CHD participating in the Framingham Offspring Study [14].

Besides their role in RCT, HDL particles exert their antiatherosclerotic role through several other mechanisms, such as a reduction of inflammation, endothelial dysfunction, and LDL oxidation [15]. Thus, proteins involved in the inflammatory response, such as serum amyloid A (SAA), have been located in specific HDL subspecies [16–18].

Several inflammatory markers and adipokines have been subjected to intensive studies for their role in insulin resistance and atherosclerosis. In particular, high-sensitivity C-reactive protein (hsCRP) and interleukin-6 (IL-6) have been clearly involved in both insulin resistance and atherosclerosis prediction [19–24]. Despite the growing body of evidence indicating that determination of HDL subpopulations may add important information on CHD risk [5, 10], data on the potential role of different HDL subpopulations in the inflammatory process are still limited [25]. This information may be particularly valuable in type 2 diabetic women, whose HDL particles are typically dysfunctional, as we have recently demonstrated [14].

In that same population of CHD-free women with and without type 2 diabetes [14], we now further investigate the potential relationships between different HDL LpA-I and LpA-I:A-II subclasses and markers of systemic inflammation.

## 2. Methods

### 2.1. Study Subjects

Study population has been previously described elsewhere [14]. Briefly, eighty type 2 diabetic and 80 nondiabetic women were consecutively recruited among those attending the metabolic disease outpatient clinic of Messina University Hospital and from voluntary employees of the same institution. The two groups were matched for age (age range: 40–62 years) and menopause (37 pre- and 43 postmenopausal in each group).

Exclusion criteria for all participants were as follows: pregnancy, hormonal replacement therapy, oral contraceptive use or multivitamin supplementation, current treatment with *β*-blockers, fibrates, statins, omega 3 fatty acids, niacin, or anti-inflammatory drugs, fasting serum creatinine >1.5 mg/dL (>132.7 *μ*mol/L), macroalbuminuria (Albustix positive), any major medical condition in the last 6 months preceding the study, and documented cardiovascular disease (CVD, defined as myocardial infarction, ischemic heart disease, coronary heart bypass, coronary angioplasty, cerebral thromboembolism, and peripheral amputations).

Lifestyle and clinical data were collected through a standardized questionnaire.

BMI and blood pressure (BP) were measured according to standard procedures. Type 2 diabetes was diagnosed according to ADA criteria [26]. Diabetic women had a mean duration of disease of 5.7 ± 6.7 years and a mean HbA_1c_ of 7.4 ± 1.5%. Subjects participating in the study were on the following diabetes therapies at enrolment: 4 (5.0%) were taking sulfonylureas alone, 24 (30.0%) were taking metformin alone, 32 (40.0%) were taking a combination of metformin and sulfonylureas, 8 (10.0%) were taking repaglinide, 2 (2.5%) were on insulin in combination with metformin and sulfonylureas, and 10 (12.5%) were not taking any medication for diabetes. None of the participants was on acarbose, glitazones, and/or incretins at the time of the study. Retinopathy was diagnosed in 15% of diabetic participants and 5% of them had microalbuminuria.

All the participants gave their informed consent and the study was approvedby the local ethical committee.

### 2.2. Biochemical Analyses

After a 12- to 14-hour fasting, blood samples were collected from all participants for the determination of the study parameters. Blood was drown in a 10 mL tube containing EDTA (0.15% final concentration) and in a regular 10 mL tube. After collection, plasma and serum were immediately separated at 2,500 rpm for 30 minutes at 4°C, and aliquots were stored at −80°C until analysis. Fasting plasma glucose and serum creatinine levels were measured with standard automated laboratory methods (Roche Diagnostics, Milan, Italy). Glycated haemoglobin (HbA_1c_) was measured using an automated high-performance liquid chromatography (HPLC) analyzer (Diamat: Bio-Rad Laboratories, Milan, Italy); normal range values in our laboratory are 4–6%. Fasting insulin concentration was measured by radioimmunoassay (Diagnostic Corporation, LA, CA, USA).

### 2.3. Plasma Lipids, Lipoprotein, and HDL Subpopulation Measurements

All lipid and lipoprotein measurements were performed at the Lipid Metabolism Laboratory, Tufts University. Plasma total cholesterol (TC) and triglycerides levels were measured by automated enzymatic assays [27]. Direct low-density lipoprotein cholesterol (LDL-C) was measured with reagents from Equal Diagnostics (Exton, PA). HDL cholesterol (HDL-C) was measured directly with a kit from Roche Diagnostics (Indianapolis, IN). Very-low-density lipoprotein cholesterol (VLDL-C) was calculated with the following equation: VLDL-C – TC − (LDL-C + HDL-C).

Apo-A-I containing HDL subpopulations in plasma were measured by nondenaturing two-dimensional gel electrophoresis, as previously described [5]. Briefly, HDL were first separated by charge, on agarose gel, into pre-*β*, *α*, and pre-*α*-mobility particles. In the second dimension, each of these 3 fractions of HDL was further separated according to size (into pre*β*1 and 2, *α*1, 2, and 3, and pre*α*-1, 2, and 3) by nondenaturing polyacrylamide gel electrophoresis. This was followed by transfer into a nitrocellulose membrane and immunoblotting with a monospecific anti-Apo-A-I primary antibody and a ^125^I-labelled secondary antibody. Signals were quantitated by image analysis using a FluoroImager (Molecular Dynamics, Sunnyvale, CA). Apo-A-I concentrations of the subpopulations were calculated by multiplying the percent of each subpopulation by the plasma total Apo-A-I concentration. The CV was <10% for *α* particles and was <15% for all other subpopulations.

### 2.4. Markers of Systemic Inflammation

All inflammatory markers measurements were performed at the Lipid Metabolism Laboratory, Tufts University. Measurements of hsCRP were performed on a Hitachi 911 (Roche Diagnostics, Indianapolis, Indiana) using the hsCRP kit from Wako Chemicals. Within- and between-run coefficients of variation were <5%. Plasma concentrations of interleukin- (IL-) 6 were determined by an ELISA assay (R&D Systems, Minneapolis, Minnesota).

### 2.5. Statistical Analysis

The numerical data are expressed as mean and standard deviation (SD). Examined variables were normally distributed as verified by Kolmogorov-Smirnov test; consequently, the parametric approach has been used. For each parameter, we performed statistical comparisons between women with and without diabetes applying Student's* t*-test. The Pearson correlation test was applied in order to assess the existence of significant interdependence between hsCRP and all numerical parameters, as well as IL-6 and all numerical parameters.

Finally, linear regression models were estimated in order to assess the possible dependence of hsCRP on all examined variables; firstly, we estimated all univariate models; subsequently, a multivariate regression analysis was performed including in the model only the variables significantly associated with inflammatory markers levels in the univariate approach. The same analysis was performed in order to assess the dependence of IL-6.

*P* < 0.05 was considered to be statistically significant.

Statistical analysis was performed using the SPSS program, version 11.0, for Windows (SPSS Inc., Chicago, IL).

## 3. Results

### 3.1. Lipid Profile, Apo-AI Containing HDL Subpopulations Distribution, and Markers of Systemic Inflammation in Women with and without Type 2 Diabetes

Clinical characteristics of the 160 CHD-free women, 80 with and 80 without type 2 diabetes, participating in the study have been previously described [14] and are shown in Table 1. Women participating in the study were matched for age, menopausal status, and menopause duration. Overall, type 2 diabetic women had higher BMI and waist circumferences, systolic and diastolic BP, and fasting plasma glucose than nondiabetic ones (*P* < 0.001 for all comparisons). These differences remained statistically significant after adjustment for age and BMI (Table 1).

As shown in Table 1, plasma concentration of triglycerides was higher (*P* = 0.001; age- and BMI-adjusted *P* < 0.05) and levels of HDL-C (*P* < 0.0001, also after age- and BMI-adjustment), Apo-AI (*P* = 0.04; not significant after adjustment for age and BMI), and Apo-AII (*P* = 0.01; age- and BMI-adjusted *P* < 0.05) were lower in diabetic women than in nondiabetic women.

When comparing circulating levels of principal Apo-AI containing HDL subpopulations (Table 1), *α*-1 (*P* = 0.006; age- and BMI-adjusted *P* < 0.05), *α*-2 (*P* = 0.005; age- and BMI-adjusted *P* < 0.05), and pre-*α*-1 HDL (*P* = 0.02; age- and BMI-adjusted *P* < 0.05) were significantly lower and *α*-3 HDL (*P* = 0.02; age- and BMI-adjusted *P* < 0.05) levels were significantly higher in diabetic women than in control women.

Diabetic women also had 2-fold higher hsCRP serum levels than nondiabetic ones (age- and BMI-adjusted *P* < 0.001); similarly, also IL-6 serum levels (age- and BMI-adjusted *P* < 0.001) were higher in diabetic women than in control women.

### 3.2. Correlations of Serum Levels of Markers of Systemic Inflammation with Metabolic Parameters, Lipid Profile, and Apo-AI Containing HDL Subpopulations Distribution in Women with and without Type 2 Diabetes

Overall, markers of systemic inflammation significantly correlated with metabolic and lipid parameters and HDL subpopulations. In particular, hsCRP levels positively correlated with IL-6 in both the diabetic group (*r* = 0.50; *P* < 0.001) and the control group (*r* = 0.55; *P* < 0.001).

As shown in Table 2, circulating hsCRP and IL-6 significantly correlated with BMI, waist circumference, fasting blood glucose, and insulin levels (*P* < 0.05 for all); IL-6 correlated with age and systolic and diastolic BP.

In the whole study population, both hsCRP and IL-6 positively correlated with triglycerides and inversely correlated with HDL-C and Apo-AII concentrations; IL-6 also showed inverse correlation with Apo-AI levels (*P* < 0.05 for all).

Significant correlations of inflammatory markers with specific Apo-AI containing HDL subclasses were also noted. Notably, hsCRP and IL-6 showed significant inverse correlations with the larger lipid-rich *α*-1, *α*-2, and pre-*α*-1 HDL subclasses and a positivecorrelation with the smaller, lipid-poor *α*-3 HDL particles. In particular, hsCRP inversely correlated with *α*-1 (*P* = 0.01) and pre-*α*-1 (*P* = 0.003); IL-6 negatively correlated with *α*-1 (*P* = 0.003), *α*-2 (*P* = 0.004), and pre-*α*-1 (*P* = 0.002) and positively with *α*-3 (*P* = 0.03).

Similar correlations were also noted when separately considering diabetic women and controls, although these correlations were less numerous, especially in controls (Table 2).

In particular, in women with diabetes, hsCRP significantly correlated with BMI (*P* < 0.001), waist circumference (*P* < 0.001), and fasting insulin (*P* < 0.001) and negatively correlated with creatinine (*P* = 0.025); moreover, hsCRP levels showed a significant correlation also with triglycerides (*P* = 0.012) and an inverse correlation with HDL-C (*P* = 0.018) and Apo-AII (*P* = 0.037) and with *α*-1 (*P* < 0.05) and pre-*α*-1 HDL subclasses (*P* < 0.05). IL-6 levels significantly correlated with BMI (*P* = 0.002), systolic (*P* = 0.036) and diastolic BP (*P* = 0.018), and fasting insulin (*P* < 0.001) and negatively correlated with Apo-AII (*P* = 0.016) and *α*-2 HDL subclasses (*P* = 0.018).

In women without diabetes, hsCRP levels showed a significant correlation with BMI (*P* < 0.001), waist circumference (*P* < 0.001), fasting blood glucose (*P* = 0.029), and fasting insulin (*P* < 0.001), whereas no significant correlation was noted with lipid profile or HDL subfractions. IL-6 levels significantly correlated with age (*P* = 0.035), waist circumference (*P* = 0.018), systolic BP (*P* = 0.038), and fasting insulin (*P* < 0.001) and inversely correlated with Apo-AII (*P* = 0.025) and *α*-1 HDL subclasses (*P* = 0.034); the correlation of BMI with IL-6 was more significant in controls than in diabetic women (*P* < 0.001).

### 3.3. Univariate and Multivariate Regression Analysis between hsPCR and IL-6 Levels and Metabolic, Lipid, and Apo-AI HDL Particles Profile in Total Study Population

At univariate regression analysis (Table 3), diabetes, BMI, waist circumference, fasting blood glucose, and insulin levels were the factors significantly associated with hsCRP concentrations in the whole study population. Circulating hsCRP was also inversely associated with HDL-C and Apo-AII concentrations and with *α*-1 and pre-*α*-1 HDL particles.

IL-6 was significantly associated with BMI, waist circumference, systolic BP, fasting blood glucose, and insulin levels and negatively with HDL-C, Apo-AI, and Apo-AII levels. A trend was also noted for an association with diabetes. IL-6 also showed significant associations with almost all the HDL subclasses explored, specifically a negative association with *α*-2 and pre-*α*-1 particles and a positive association with *α*-3 HDL subfractions.

Multivariate regression analysis was performed including in the model only HDL subpopulations and not HDL-C levels, to avoid colinearity. As a result (Table 3), BMI was the only factor significantly associated with hsCRP concentrations in the whole study population. However, a trend was noted for an inverse association of hsPCR levels with pre-*α*-1 HDL particles. BMI and fasting plasma glucose were significantly associated with IL-6 levels, whereas no significant association was described with lipid profile.

## 4. Discussion

Low levels of HDL-C are a mainstay of diabetic dyslipidemia and a largely recognized CHD risk factor [28–30], especially in insulin resistant patients [31].

HDL particles may be particularly atheroprotective in women, where each 1 mg/dL increase in HDL-C is associated with a 3% decrease in CHD risk versus 2% in men [6].

The antiatherosclerotic role of these particles may be also mediated by the modulation of inflammation, since atherosclerosis today is considered an inflammatory disease.

We have recently shown that diabetic women have a less atheroprotective HDL subpopulation pattern [14]. In this study, we investigated the potential relationship of HDL-C levels, HDL subclasses, and hsPCR and IL-6 levels, two well-known markers of inflammation, in that cohort of CHD-free women with and without type 2 diabetes [14].

Both hsCRP and IL-6 are well-characterized inflammatory markers in type 2 diabetes [19–24], being independently related to insulin resistance [32] and to the progression of atherosclerosis [33]. Women usually have higher hsCRP levels than men [34, 35], probably as a consequence of their relatively higher degree of visceral adiposity. In our female study population, both hsCRP and IL-6 levels were higher in diabetic women than in controls, although this difference was statistically significant only for hsCRP. These observations are largely consistent with previous studies showing a high degree of subclinical inflammation in the presence of diabetes [25, 36]. Furthermore, gender differences were also reported in these associations. Thus, in the Mexico City Diabetes Study [37], hsCRP levels were associated with incident diabetes in women but not in men, and in the MONICA/KORA study the association of hsCRP with the risk of type 2 diabetes was stronger in women [38].

Our results also confirm the association of inflammatory markers with adiposity and insulin resistance, since BMI, waist circumference, fasting blood glucose and insulin, and systolic and diastolic BP were all significantly associated with inflammatory markers, especially in women with diabetes. However, these associations are probably driven by the deleterious effects of obesity, since at multivariate analysis BMI was the strongest correlate of inflammatory markers in our dataset, even more than diabetes itself, which was no longer significant at multivariate analysis, although fasting blood glucose was still significantly associated with IL-6 levels.

When the potential relationships between inflammation and lipid profile, with particular regard to HDL particles, were assessed, we found significant correlations between inflammatory markers and HDL-C levels and Apo-AI and Apo-AII concentrations, especially in diabetic women, whereas no associations were noted with other lipid fractions. Although many of these correlations disappeared when separating women with and without diabetes, probably because of the smaller sample size, these results are in accordance with those of the ATTICA study, where a significant correlation between HDL-C concentrations and markers of systemic inflammation was shown [25]. Accordingly, familial low-HDL-C subjects display higher levels of hsCRP [39].

The potential anti-inflammatory role of HDL particles is sustained by several lines of evidence. Thus, besides their role in RCT, HDL particles may have several other antiatherosclerotic mechanisms, including the modulation of oxidation, inflammation, and endothelial dysfunction [40].

Indeed, as reported in numerous studies [41, 42], HDL may stimulate nitric oxide and prostacyclin production from endothelial cells, regulate vascular structure and tone, promote endothelial survival [43], and influence immunity, modulating the expression of inflammatory chemokines and complement system [44]. Furthermore, the numerous enzymes carried by these lipoproteins, such as paraoxonase, platelet-activating factor-acetyl hydrolase, LCAT, or glutathione seleno peroxidase, prevent LDL oxidation and confer HDL anti-infectious properties [44, 45].

The anti-inflammatory properties of HDL particles have been also sustained by proteomics analysis, revealing more than 50 proteins associated with HDL, most of which are with specific anti-inflammatory or antioxidant functions.

However, the link between inflammation and HDL particles is complex. Thus, the protective role of HDL-C levels appears to be attenuated by acute or chronic inflammation [46].

*In vitro* studies have shown that HDL isolated from coronary artery disease (CAD) subjects are able to exert proinflammatory properties when compared to particles isolated from controls [47].

Anti-inflammatory effects of HDL particles may be also particularly relevant in acute coronary syndrome (ACS), where vascular inflammation strongly affects plaque vulnerability [48]. Thus, a significant shift in the HDL proteome of ACS subjects was observed, with modifications in several proteins including Apo-AIV, C3 complement, HDL-associated haemoglobin, and SAA [49]. Thus, the apoprotein and enzyme constituents of HDL can be replaced by acute phase reactants (serum amyloid A, fibrinogen), which attenuates the capacity of HDL to mediate other antiatherogenic functions [49]. However, whether the “inflammatory” state of HDL is able to impair their ability in RCT is still a matter of debate [49, 50].

These and other experimental lines of evidence indicate that, under inflammatory conditions, HDL particles lose their protective capacity shifting toward a proatherogenic pattern [51, 52], probably because of HDL remodelling, leading to modifications in composition and structure of HDL particles [51, 53, 54]. All these lines of evidence suggest the necessity of determining the “quality” of HDL particles more than estimating their quantity [5, 55], a concept that has led some authors to define an “inflammatory index” to quantify the pro- or anti-inflammatory profile of HDL [56, 57].

It is becoming apparent that different HDL particles may show peculiar “qualities” that may influence RCT process, as well as their antioxidant or anti-inflammatory potential, rendering them atheroprotective or proatherogenic [58].

In CHD patients, Asztalos et al. showed distinct alterations in HDL subpopulation distribution, as assessed by nondenaturing two-dimensional electrophoresis [5]. Accordingly, in our group of CHD-free type 2 diabetes women, we previously observed these same alterations in HDL subpopulation distribution, with a reduction of large lipid-rich *α*-1, *α*-2, and pre-*α*-1 HDL and an increase of the small, lipid-poor *α*-3 HDL subpopulations [14].

Since these modifications could negatively influence anti-inflammatory properties of HDL particles, we also tested the hypothesis that different HDL LpA-I and LpA-I:A-II subclasses may be differently associated with inflammation. Our data confirm this hypothesis, since markers of inflammation negatively correlated with large lipid-rich *α*-1 and *α*-2 HDL subfractions, which are considered more atheroprotective. IL-6 levels also positively correlated with the small *α*-3 HDL concentrations, which show proatherogenic properties. These correlations were more evident in women with diabetes. Notably, low levels of *α*-1 HDL particles have been shown to be the most significant predictor of recurrence of cardiovascular events in CHD patients [59], and the negative association of this HDL subfraction with hsCRP levels observed in our study suggests that the modulation of inflammation may play a crucial role.

Although the small sample size is a limitation, in our study population, the use of lipid-lowering medications, anti-inflammatory drugs, and glitazones was accurately excluded to avoid their confounding effect on the relationship between inflammatory markers and lipid variables.

Another limitation is the cross-sectional design of our study that does not allow us to determine whether a specific HDL profile is less “anti-inflammatory” or, on the contrary, it is the higher inflammatory state which modifies HDL particles distribution toward a proatherogenic pattern.

In conclusion, our data show that HDL-C and the more atheroprotective HDL subpopulations are inversely associated with inflammatory markers, suggesting that different HDL particles may exert a different role in inflammation. However, caution must be taken when interpreting these associations that need to be confirmed in larger populations.

The functionality of HDL particles is a matter of growing investigation and, while waiting for validated markers in the clinical practice, the measurement of specific HDL subfractions might be useful to better evaluate the CVD risk in diabetic subjects.

## Figures and Tables

**Table 1:** Lipid profile, Apo-AI containing HDL subpopulations distribution, and markers of systemic inflammation in women with and without type 2 diabetes.

	Total population	Women with type 2 diabetes	Women without type 2 diabetes	*P*
*n*	160	80	80	
Postmenopausal (*n*)	86	43	43	
Age (yrs)	51.32 ± 10.13	52.03 ± 9.70	50.61 ± 10.56	—
Menopausal duration (yrs)	9.04 ± 7.62	8.59 ± 6.67	9.35 ± 8.27	—
BMI (Kg/m^2^)	29.52 ± 6.85	32.38 ± 6.91	26.47 ± 5.34	<0.001
Waist circumference (cm)^§^	95.46 ± 14.28	99.71 ± 12.58	88.67 ± 14.34	<0.001
Systolic BP (mmHg)∗	126.79 ± 16.33	131.5 ± 18.01	121.84 ± 12.69	<0.001
Diastolic BP (mmHg)^§^	75.58 ± 9.16	78.75 ± 8.88	72.24 ± 8.26	<0.001
Fasting BG (mg/dL)∗	129.02 ± 46.23	160.54 ± 47.16	97.11 ± 5.22	<0.001

Lipid and lipoprotein profile				
Total-C (mg/dL)^#^	191.81 ± 29.32	190.86 ± 29.44	192.75 ± 29.36	—
LDL-C (mg/dL)^#^	124.97 ± 27.82	124.46 ± 27.23	125.5 ± 28.60	—
Triglycerides (mg/dL)^§^	104.50 ± 63.37	120.7 ± 78.2	88.2 ± 37	0.001
HDL-C (m/dL)∗	51.78 ± 13.40	47.35 ± 13.58	56.22 ± 11.71	<0.0001
Apo-AI (mg/dL)^#^	125.94 ± 19.78	122.75 ± 20.69	129.14 ± 18.34	0.04
Apo-AII (mg/dL)^§^	31.09 ± 4.82	30.14 ± 5.24	32.04 ± 4.18	0.01

Apo-AI containing HDL subpopulations profile				
*α*-1 HDL (mg/dL)^§^	21.33 ± 9.47	19.32 ± 8.97	23.35 ± 9.58	0.006
*α*-2 HDL (mg/dL)^§^	43.38 ± 9.52	41.29 ± 9.62	45.47 ± 8.99	0.005
*α*-3 HDL (mg/dL)^§^	17.27 ± 4.81	18.18 ± 5.56	16.36 ± 3.74	0.02
Pre-*α*-1 (mg/dL)^§^	6.13 ± 3.47	5.51 ± 3.39	6.74 ± 3.46	0.025

Markers of systemic inflammation				
hsCRP (mg/L)∗	4.31 ± 6.34	5.93 ± 7.66	2.68 ± 4.11	0.001
IL-6 (pg/mL)∗	2.30 ± 2.69	2.70 ± 3.23	1.9 ± 1.94	—

Data are *n*, means ± SD. Only significant *P* values for the comparisons between diabetic and nondiabetic women are presented. Total-C: total cholesterol; Apo: apolipoprotein. ∗Age- and BMI-adjusted *P* value < 0.001; ^§^age- and BMI-adjusted *P* value < 0.05; ^#^nonsignificant age- and BMI-adjusted *P* value.

**Table 2:** Correlation coefficients (*r*_*S*_) between markers of systemic inflammation and metabolic, lipid, and Apo-AI containing HDL subpopulations profile in women with and without type 2 diabetes.

	Total population		Women with type 2 diabetes		Women without type 2 diabetes	
	hsCRP	IL-6	hsCRP	IL-6	hsCRP	IL-6
Age	—	0.17∗	—	—	—	0.24∗
Menopause duration	—	—	—	—	—	—
BMI	0.62^§^	0.48^§^	0.55^§^	0.35∗	0.58^§^	0.49^§^
Waist C	0.57^§^	0.37^§^	0.45^§^	—	0.53^§^	0.35∗
Systolic BP	—	0.30^§^	—	0.24∗	—	0.24∗
Diastolic BP	—	0.29^§^	—	0.27∗	—	—
Fasting BG	0.35^§^	0.35^§^	—	—	0.25∗	—
Fasting insulin	0.51^§^	0.43^§^	0.45^§^	0.36^§^	0.52^§^	0.44^§^
Creatinine	—	—	−0.25∗	—	—	—

Lipid and Apo-AI containing HDL subpopulations profile						
Total-C	—	—	—	—	—	—
LDL-C	—	—	—	—	—	—
Triglycerides	0.28^§^	0.19∗	0.28∗	—	—	—
HDL-C	−0.23∗	−0.29^§^	−0.26∗	—	—	—
Apo-AI	—	−0.19∗	—	—	—	—
Apo-AII	−0.19∗	−0.32^§^	−0.23∗	−027∗	—	−0.25∗
*α*-1 HDL	−0.19∗	−0.24∗	−0.22∗	—	—	−0.24∗
*α*-2 HDL	—	−0.33∗	—	−0.27∗	—	—
*α*-3 HDL	—	0.18∗	—	—	—	—
Pre-*α*-1 HDL	−0.023∗	−0.24∗	−0.21∗	—	—	—

Only significant correlation coefficients (Spearman test) are shown. ^§^ *P*, *P* value < 0.001; *P, *P* value < 0.05. Waist C: waist circumference; BP: blood pressure; BG: blood glucose, Total-C: total cholesterol; Apo: apolipoprotein.

**Table 3:** Univariate and multivariate regression analysis between hsCRP and IL-6 and metabolic, lipid, and Apo-AI containing HDL subpopulations profile in total population.

	hsPCR				IL-6			
	Univariate regression		Multivariate regression		Univariate regression		Multivariate regression	
	*B*	*P*	*B*	*P*	*B*	*P*	*B*	*P*
Anthropometric and metabolic parameters								
Diabetes	3.251	0.001	—	—	0.800	0.061	—	—
BMI	0.41	<0.001	0.24	0.03	0.14	<0.001	0.141	0.003
Waist C	0.11	0.001	—	—	0.03	0.02	—	—
Systolic BP	—	—	—	—	0.03	0.01	—	—
Fasting BG	0.03	0.005	—	—	0.01	0.009	0.011	0.02
Fasting insulin	0.09	0.004	—	—	0.04	0.009	—	—

Lipid and Apo-AI containing HDL subpopulations profile								
HDL-C	−0.10	0.005	—		−0.05	0.002	—	
Apo-AI	—	—	—	—	−0.03	0.003	—	—
Apo-AII	−0.21	0.04	—	—	−0.13	0.004	—	—
*α*-1 HDL	−0.11	0.04	—	—	—	—	—	—
*α*-2 HDL	—	—	—	—	−0.06	0.009	—	—
*α*-3 HDL	—	—	—	—	0.11	0.04	—	—
Pre-*α*-1 HDL	−0.39	0.007	−0.34	0.083	−0.13	0.03	—	—

Only significant *P* are presented. Waist C: waist circumference; BP: blood pressure; BG: blood glucose; Apo: apolipoprotein.
